# Rights of an unborn baby versus the social and legal constraints of parents: Birth of a new debate

**DOI:** 10.4103/0971-9261.43788

**Published:** 2008

**Authors:** Ravi Kanojia

**Affiliations:** Department of Pediatric Surgery, PGIMER, Chandigarh - 160012, India. E-mail: doctor.ravi@yahoo.com

Like everyone else we had been hearing about the recent Mumbai abortion case through the media. As Pediatric Surgeons and sensitive human beings we all felt quite disturbed over various aspects of the issue. The case raised a debate regarding the rights of an unborn baby and those of its parents.

A 30-year-old mother, had a 24-week pregnancy. The baby was diagnosed to have a congenital heart disease. Knowing about the morbidity related to the disease, the couple decided not to have the baby; however, the pregnancy was already well beyond 20 weeks. They asked for legal permission from the court for abortion. The court denied it as it was against the law. The case ended with the twisted tale of some wrong reports from the hospital and with the miscarriage possibly because of the stress the mother had undergone. The event definitely left us with some food for our thoughts.

A nationwide debate followed, with the majority of the learned politicians, professionals, lawyers and doctors arguing against the abortion. However, a significant proportion of the general public did think that parents should be allowed to decide whether to have a defective baby or not, as they are the people who will face the ground realities and not the society or the system who is forcing them to keep the baby.

There was a very noticeable fact that many categories of people were being asked for their opinion (lawyers, IMA president, CTVS surgeons, etc.). These people saw the issue in their limited perspectives. We never saw any one asking any pediatric surgeon about what is probably scientifically correct. We feel that pediatric surgeons, after gynecologists, are the people directly related to facing situations where a mother comes with an antenatal ultrasound past 20 weeks with congenital defects in the baby. It would not be wrong to say that a pediatric surgeon is the most authentic person to give his opinion regarding the morbidity associated with a congenital anomaly. We see and analyze the situation in a much wider perspective while considering a whole lot of other congenital anomalies being increasingly diagnosed antenatally, many of which prove to be incompatible with life or associated with poor quality of life. The family and the society have to bear the severe burden of these babies later on.

## WHAT DOES THE LAW SAY?

The 1971 MTP act specifically mentions laws regarding:

- When a pregnancy can be terminated.
- By whom it can be terminated.
- Place where pregnancy can be terminated.
- The punishments of violation.

The major point of discussion in relation of these laws is that no pregnancy can be terminated after 20 weeks have elapsed in any circumstance unless there is a life-threatening medical emergency to the mother, like in a situation of a threatened abortion. Hence, even if a mother is diagnosed to have a baby with anencephaly or multiple defects incompatible to life after birth, she will be forced to keep the baby once the time limit of 20 weeks has been crossed.

## HOW FAR HAVE WE COME ALONG AFTER FORMULATING THE LAW, i.e. THE AMENDMENTS?

After enactment of the law in 1972, a few amendments were made that were minor ones and changed the definition of a mentally ill person versus a lunatic, change in the places authorized for MTP, the equal liability of the owner of the place where MTP is performed even if he is not the doctor and the degree of punishment after violation.

## WHAT LAW EXISTS IN OTHER COUNTRIES FOR SIMILAR SITUATIONS?

The UK Abortion Act 1967: Much of the Indian law was copied from the UK Abortion Act 1967, which included a similar time limit of 20 weeks at the time of enactment, but, with the changing scenario, the act was amended in 1990 and the time limit was increased to 24 weeks.

The exact text from the law amended is quoted below (From - http://www.efc.org.uk/Foryoungpeople/Factsaboutabortion/MoreonUKabortionlaw).

The changes made to the Abortion Act 1967 by section 37 of the Human Fertilization and Embryology Act 1990 came into effect on April 1, 1991, and included a time limit of 24 weeks for abortions under statutory grounds.

Rest of the world (text quoted from the source http://en.wikipedia.org/wiki/Abortion_law)

As of 2000, among the 152 most populous countries, 54 either banned abortion entirely on religious beliefs or permitted it only to save the life of the pregnant woman. In contrast, another 44 banned late-term abortions eg; after a particular gestational age: 12 weeks (Albania, Armenia, Azerbaijan, Belarus, Bosnia-Herzegovina, Bulgaria, Croatia, Cuba, Czech Republic, Denmark, Estonia, France, Georgia, Greece, Kazakhistan, Kyrgyz Republic, Latvia, Lithuania, Macedonia, Moldova, Mongolia, Norway, Russian Federation, Slovak Republic, Slovenia, South Africa, Ukraine, Tajikistan, Tunisia, Turkey, Turkmenistan, Uzbekistan and Yugoslavia), 13 weeks (Italy), 14 weeks (Austria, Belgium, Cambodia, Germany, Hungary and Romania), 18 weeks (Sweden), viability (Netherlands and to some extent the United States) and 24 weeks (Singapore and the United Kingdom). Here, the term viability is equivalent to 24 weeks.

### Our view point (debatable)

1. Liberalize the time limit to 24 weeks instead of 20.
2. Develop a categorical list of congenital anomalies that are either:
  - incompatible with life,
  - carry extreme morbidity after birth or
  - keep the individual away from a socially productive life.
  Any of these listed anomalies if found at any stage of pregnancy, the parents should have the right to legally terminate.
3. Legal provisions making advanced-level antenatal ultrasound mandatory (which is to be made available free of cost by the state if possible).

## WHAT DO THE LEGISLATORS FEAR IF THE LAW IS LIBERALIZED?

There were many arguments against the liberalization of the law in such circumstances as seen in the Mumbai abortion case. Many of them were valid. It will result in killing of the unborn baby, specially the girl child. But again, there are both good and bad aspects of the situation. All these can be controlled by strict law enforcement and monitoring agencies like the PNDT Act and the government health departments.

## WHAT ARE THE GROUND REALITIES?

We are all aware that the health care scenario for an average citizen is not optimal in India and is in a grim situation. Even the most basic antenatal health care program does not receive priority over other health schemes. In this situation, it is almost impossible to provide good antenatal screening services for congenital anomalies. In the absence of a state-sponsored health insurance, the entire cost of treatment has to be borne by the parents. This stands in contrast to the developed countries like the UK and the USA. Perhaps the law makers, politicians and all the people who were giving their opinion in the mentioned case should keep this in mind and think with a wider perspective of the field situation in our country. Even the judge, while giving the final decision, said that “There was no law in place for the court to order termination of the pregnancy in a situation like this. The government can consider making this change in the future, but what can we do now?” This meant that while giving this decision even the judges realized that what they were giving was not fair and changes need to be made.

## ROLE OF IAPS

We have a strong association in the form of IAPS, which can be verbose enough to deliver authentic opinions on such issues. We have to speak up with both the ground realities as well as the scientific facts. The only way to jump into the scene and produce significant contributions is by constituting a scientific committee that can study the situation and convey the key message to the law makers.

The above text deliberately leaves several aspects of the agenda untouched, perhaps to provoke “us,” who will bring our thoughts from passive subconsciousness to active and executable decisions after reading this. In the end, we can say that change with time is a rule and even the rules have to change with time in order to remain valid in the most current context. We made a rule 38 years back and did not make any major change in it, a thing that other countries did as they progressed with time.

